# Multispectral UAV dataset for multi-temporal mangrove monitoring in the Cirebon Coastal Area, Indonesia (2025–2026)

**DOI:** 10.1016/j.dib.2026.113346

**Published:** 2026-10-12

**Authors:** Umar Abdurrahman, Mega Laksmini Syamsuddin, Subiyanto, Ismail Maqbul, Mochamad Rudyansyah Ismail, Athaurrahman Dzaky Wattiheluw, Muhamad Faqih Hidayatullah, Indrawan Fadhil Pratyaksa, Diyah Fatimah Oktaviani, Ivonne Milichristi Radjawane

**Affiliations:** a Department of Marine, Faculty of Fisheries and Marine Sciences, Universitas Padjadjaran, Jatinangor, West Java, 45363, Indonesia; b Center of Climate and Maritime Regional Management, Faculty of Fisheries and Marine Sciences, Universitas Padjadjaran, Jatinangor, West Java, 45363, Indonesia; c Agro-Industrial Product Development, Politeknik Negeri Cilacap, Cilacap, Central Java, 53212, Indonesia; d Blue Arc Global, South Jakarta, Jakarta, 12710, Indonesia; e Korea-Indonesia Marine Technology Cooperation Research Center, Cirebon Center, West Java, 45611, Indonesia; f Oceanography Study Program, Faculty of Earth Sciences and Technology, Institut Teknologi Bandung, Bandung, 40132, Indonesia

**Keywords:** Mangrove ecosystem, Multi-temporal UAV, Multispectral remote sensing, Cirebon Coastal Area, Blue carbon

## Abstract

This article presents a multi-temporal unmanned aerial vehicle (UAV) multispectral dataset acquired over mangrove conservation patches in the Cirebon Coastal Area, West Java, Indonesia (Mundupesisir), covering two survey campaigns conducted on 5 May 2025 and 12 August 2026. Both campaigns employed a DJI Mavic 3 Enterprise Multispectral UAV, capturing four-band reflectance imagery (Green, Red, Red Edge, and Near-Infrared) alongside a high-resolution Red-Green-Blue (RGB) orthomosaic. The two campaigns covered the same Mundupesisir mangrove conservation area and were georeferenced using real-time kinematic (RTK) positioning, with ground control supported by nine primary RTK Rover control points expanded with additional points for image-by-image position correction. The dataset consists of calibrated single-band multispectral reflectance rasters in GeoTIFF format and compressed RGB orthomosaic composites in Enhanced Compressed Wavelet (ECW) format, each packaged as compressed archives (.rar) per campaign, together with Exchangeable Image File Format (EXIF) metadata and RTK control point coordinates. Data were processed using Pix4D v4.7.5 with consistent processing parameters applied across both campaigns. The collection supports vegetation index derivation, change detection, and validation of satellite-based mangrove monitoring approaches.

Specifications Table

**Table:** 

Subject	Earth and Environmental Sciences – Remote Sensing; Coastal and Marine Science; Ecology and Conservation
Specific subject area	Multi-temporal unmanned aerial vehicle (UAV) multispectral dataset for mangrove ecosystem monitoring, vegetation index derivation, and coastal conservation assessment in Indonesia
Type of data	Raster images (.tif, packaged in .rar): calibrated single-band multispectral reflectance (Green, Red, Red Edge, Near-Infrared). Red-Green-Blue (RGB) composite orthomosaic (.ecw, packaged in .rar): compressed true-colour orthomosaic per campaign. Real-time kinematic (RTK) Rover control point coordinates (.csv). Exchangeable Image File Format (EXIF) metadata of aerial photographs (.txt)
Data collection	Two campaigns conducted on 5 May 2025 and 12 August 2026, using a DJI Mavic 3 Enterprise Multispectral (M3E MS) UAV; 4 spectral bands (Green 560 nm, Red 650 nm, Red Edge 730 nm, Near-Infrared (NIR) 860 nm); integrated sunlight sensor for irradiance correction. Flight altitude 60 m Above Ground Level (AGL); frontal overlap 80%, side overlap 70%; flight speed 2.9 m/s; ground sampling distance (GSD) 2.77 cm/pixel. Ground control supported by RTK Rover measurements (Leica GS18T): 9 primary control points expanded with additional points for image-by-image position correction. Processed using Pix4D v4.7.5
Data source location	Institution: Department of Marine, Faculty of Fisheries and Marine Sciences, Universitas Padjadjaran. City/Town/Region: Mundupesisir, Cirebon, West Java, Indonesia. Coordinate system: WGS84 / UTM Zone 49S.
Data accessibility	Repository Name: Zenodo Direct URL to data: https://doi.org/10.5281/zenodo.22008924 DOI: https://doi.org/10.5281/zenodo.22008924
Related research article	None

## 1 Value of the Data

• The methodological novelty of this dataset is that identical UAV platform, sensor, flight, ground-control, and processing parameters were applied across two survey dates. Nothing in the acquisition or processing chain changed between campaigns, so the two epochs can be compared directly without correcting for sensor or processing drift. Few openly available multi-temporal UAV datasets maintain this.
• The dataset covers Mundupesisir, one of the largest and most actively managed community-based mangrove conservation areas in the Cirebon Coastal Area, West Java. Openly available UAV multispectral coverage of a single managed mangrove site in this region is scarce, even though UAV platforms remain comparatively underused relative to satellite data in mangrove ecosystem assessment [1].
• Surveying the same site in May 2025 and again in August 2026 makes it possible to detect change and track mangrove canopy dynamics over time. Vegetation indices such as Normalized Difference Vegetation Index (NDVI), Normalized Difference Red Edge Index (NDRE), and Green Normalized Difference Vegetation Index (GNDVI) can be derived and compared across the two campaigns to quantify canopy health trajectories and disturbance responses, giving conservation managers measurable metrics to work from.
• The calibrated single-band reflectance rasters can be used for vegetation health assessment, species discrimination [2], and land-cover classification, among other remote sensing analyses.
• This dataset can be used as a high-resolution reference to validate and calibrate satellite-based mangrove mapping products. Resampling the UAV orthomosaics to satellite resolutions (e.g., Sentinel-2, Landsat, PlanetScope) lets researchers assess the accuracy of coarser mapping approaches — relevant to regional and national mangrove monitoring programmes in Indonesia.
• The RGB orthomosaics in compressed ECW format provide high-resolution true-colour imagery suitable for visual interpretation, digitising mangrove patch boundaries, and supporting non-specialist stakeholders such as conservation managers, local government, and community groups in monitoring and communicating mangrove status in the Cirebon Coastal Area.

## 2 Background

Mangrove ecosystems along Indonesia’s coastlines are among the most biologically productive and carbon-dense environments on Earth, yet they face persistent pressures from land-use change, aquaculture expansion, and coastal development [3]. The Cirebon Coastal Area in West Java is representative of many Indonesian coastal regions where mangrove patches persist in a fragmented, conservation-managed landscape subject to rapid urbanisation and fishery-driven land conversion. The Mundupesisir area is notable among these sites as one of the largest and most actively managed community-based mangrove conservation patches in the region, making it an important location for ongoing ecological monitoring.

Conventional field surveys are time-consuming, logistically constrained by tidal conditions, and often unable to capture the fine-scale spatial variability of mangrove canopy structure. Satellite remote sensing offers synoptic coverage but is limited by spatial resolution for patch-level analysis and by atmospheric interference and cloud cover in tropical regions. UAV-based multispectral remote sensing addresses these limitations by delivering sub-decimetre spatial resolution imagery and enabling flexible, repeatable survey campaigns at relatively low cost [4,5]. Similar UAV-based multispectral approaches have been applied to classify mangrove genera elsewhere in Indonesia, such as in the Kepulauan Seribu Islands, demonstrating the broader applicability of this method across the archipelago's mangrove ecosystems [6].

Repeated UAV surveys using consistent flight and sensor parameters allow detection of canopy changes and phenological variation that cannot be resolved from single-epoch data [7]. Multispectral UAV sensors are sensitive to chlorophyll content, leaf water status, and vegetation structural properties, particularly through their Red Edge and Near-Infrared channels. Indices derived from these bands correlate closely with mangrove canopy health [8].

This dataset was generated to support ongoing coastal ecosystem monitoring activities of the Korea-Indonesia Marine Technology Cooperation Research Center (MTCRC) in Cirebon. The two survey campaigns, on 5 May 2025 and 12 August 2026, build a temporal monitoring record for mangrove conservation patches in the Mundupesisir area.

## 3 Data Description

The repository is organised into two top-level folders corresponding to the survey campaigns: ‘2025’ and ‘2026’. Each folder contains a compressed archive of calibrated multispectral reflectance bands, a compressed archive of the RGB orthomosaic, and an EXIF metadata file. RTK Rover control point coordinates are provided as a single ancillary file applicable to both campaigns. Table 1 lists all files in the repository. Fig. 1 shows the geographical location of the survey area and UAV flight coverage footprint.

**Table 1:** Overview of dataset files by survey campaign.

Campaign	File	Contents	Format	Notes
2025-05-05	2025_MS.rar	Single-band reflectance: Green, Red, Red Edge, NIR	.tif in .rar	M3E MS, 4 bands
2025-05-05	2025_Ortho.rar	RGB true-colour orthomosaic	.ecw in .rar	Compressed composite
2025-05-05	2025_EXIF.txt	EXIF metadata of aerial photographs	.txt	Position, attitude, timestamp
2026-08-12	2026_MS.rar	Single-band reflectance: Green, Red, Red Edge, NIR	.tif in .rar	M3E MS, 4 bands
2026-08-12	2026_Ortho.rar	RGB true-colour orthomosaic	.ecw in .rar	Compressed composite
2026-08-12	2026_EXIF.txt	EXIF metadata of aerial photographs	.txt	Position, attitude, timestamp
Both	RTK_GCP.csv	RTK Rover control point coordinates	.csv	9 primary + expanded points

**Fig. 1:**
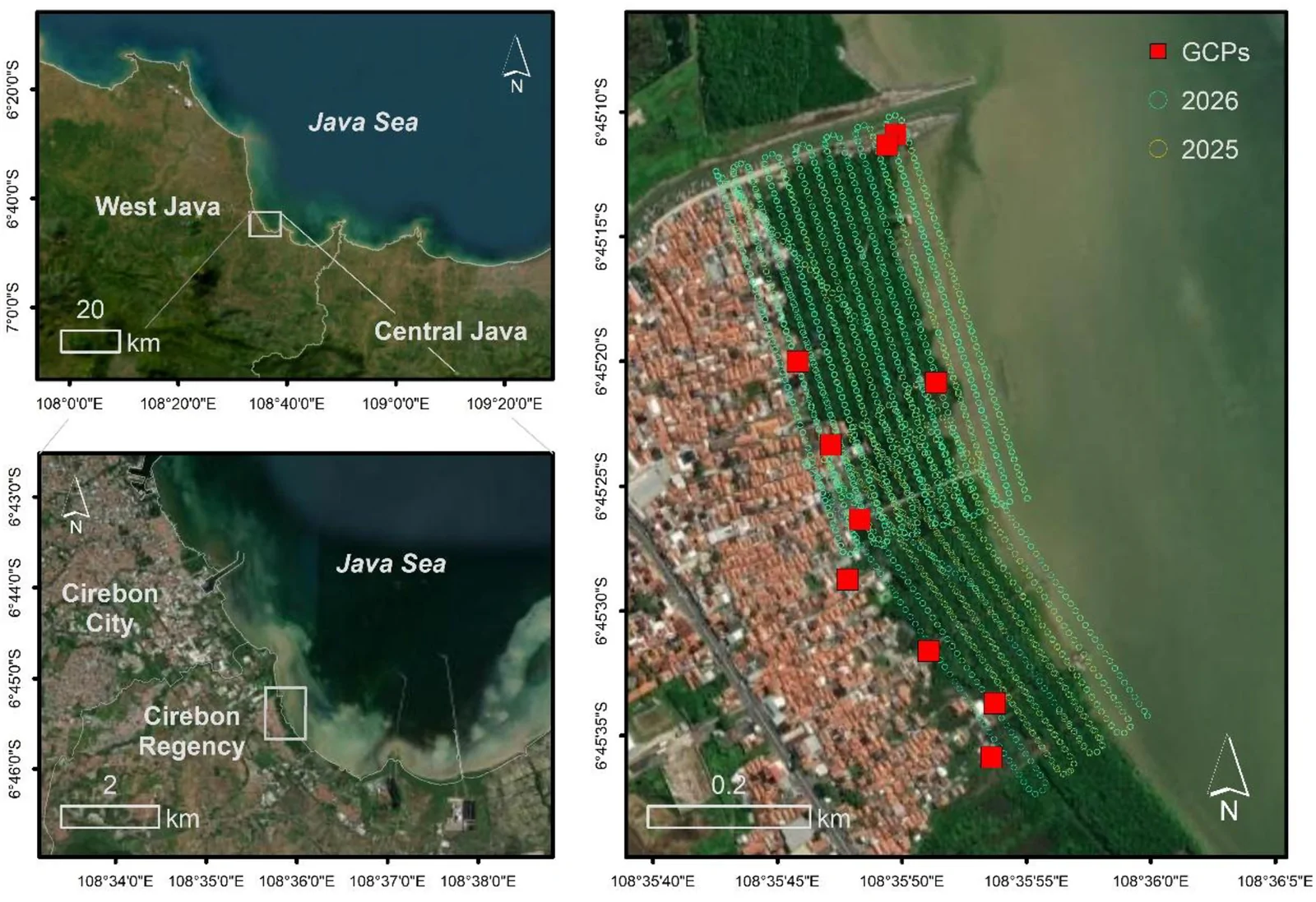
Study area and UAV flight coverage. Top-left inset: location within northern West Java. Bottom-left inset: location within the Cirebon Coastal Area. Right panel: mapping coverage over the Mundupesisir mangrove conservation area, showing ground control point (GCP) positions (red squares) and single aerial photograph positions for the 2025 and 2026 campaigns.

### 3.1 Multispectral reflectance rasters

Each campaign's primary multispectral product is a set of calibrated single-band reflectance rasters in GeoTIFF (.tif) format, packaged as a compressed archive (2025_MS.rar, 2026_MS.rar) and georeferenced in WGS84 / UTM Zone 49S. Four bands were captured for both campaigns — Green (560 nm), Red (650 nm), Red Edge (730 nm), and Near-Infrared (860 nm) — with the DJI Mavic 3 Enterprise Multispectral [9]. Pix4D v4.7.5 [10] derived the reflectance values using in-flight irradiance measurements from the integrated sunlight sensor, which normalised for the different illumination conditions on the two acquisition dates. Fig. 2 shows the derived NDVI alongside the RGB orthomosaic for each campaign.

**Fig. 2:**
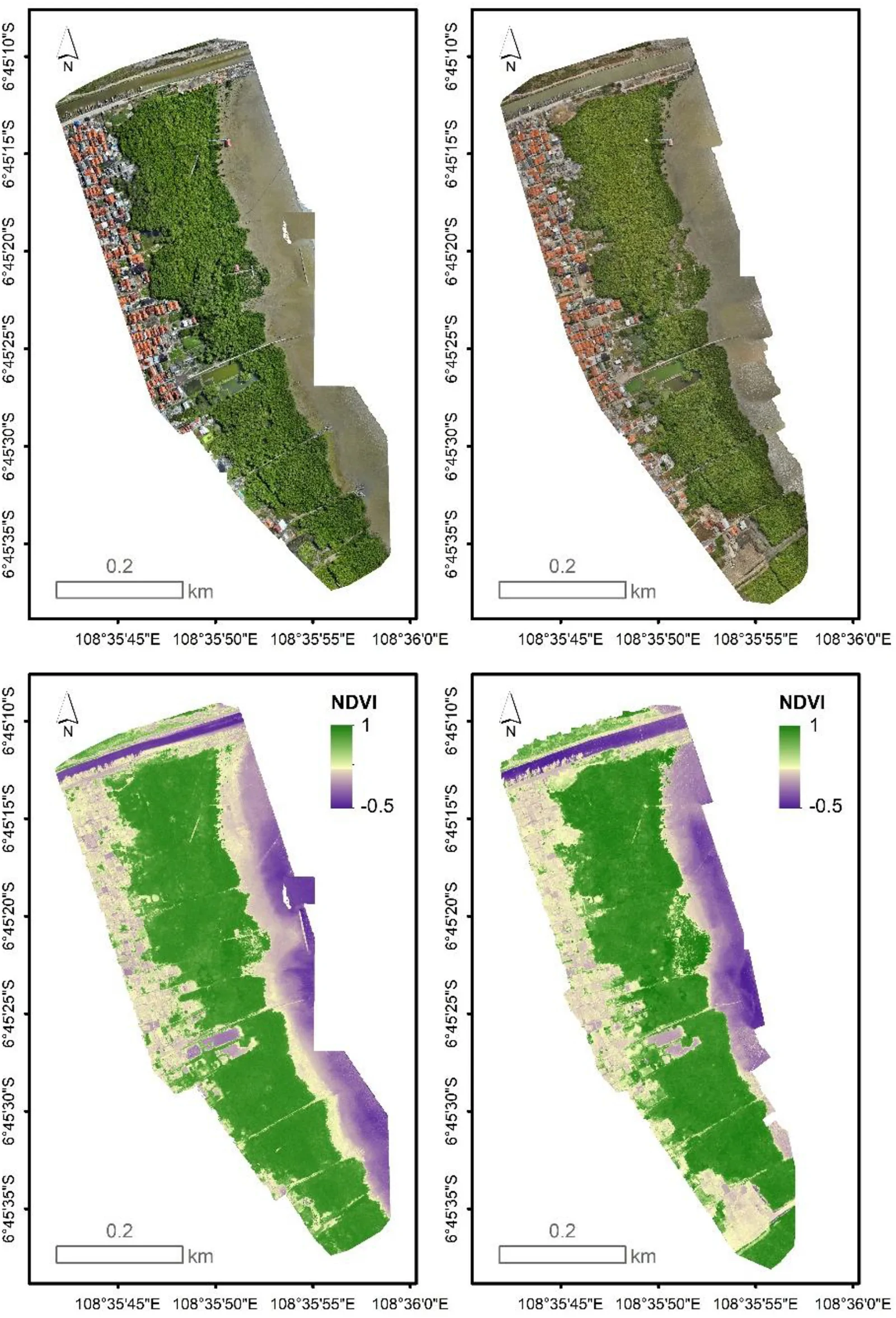
RGB orthomosaic and NDVI for the Mundupesisir mangrove conservation area. Top row: RGB true-colour composite orthomosaic. Bottom row: NDVI. Left column: 5 May 2025. Right column: 12 August 2026.

### 3.2 RGB orthomosaics

Each campaign includes a high-resolution RGB true-colour orthomosaic compressed to Enhanced Compressed Wavelet (.ecw) format and packaged as a compressed archive (2025_Ortho.rar, 2026_Ortho.rar). ECW compression cut file size without sacrificing the spatial resolution or visual quality needed for interpretation, boundary delineation, and stakeholder communication. The orthomosaics are georeferenced in WGS84 / UTM Zone 49S. Fig. 2 illustrates representative orthomosaic views of the Mundupesisir mangrove conservation area for both survey dates.

### 3.3 Ground control and positional accuracy

Ground control was established with a Leica GS18T RTK Rover [11]. Nine ground control points were surveyed across the mapped extent to support image-by-image position correction during processing. At each point the rover was held until the RTK solution reached a fixed state, after which ten epochs were logged. Position dilution of precision (PDOP) ranged from 1.0 to 1.3 across the nine points, with 9–12 GPS and 6–8 GLONASS satellites tracked. Positional accuracy averaged 0.0162 m (1D RMS), 0.0099 m (2D RMS), and 0.0190 m (3D RMS); individual point residuals ranged from 0.007 to 0.063 m (1D RMS), 0.005 to 0.037 m (2D RMS), and 0.009 to 0.073 m (3D RMS) (Table 2).

**Table 2:** Positional accuracy of the nine RTK Rover ground control points.

Metric	1D RMS (m)	2D RMS (m)	3D RMS (m)
Mean	0.0162	0.0099	0.0190
Range	0.007–0.063	0.005–0.037	0.009–0.073

## 4 Experimental Design, Materials, and Methods

### 4.1 Study area

The study area comprises mangrove stands established through staged community planting programmes rather than natural regeneration, planted incrementally by the local conservation group over multiple planting cycles. Field surveys at this site have identified four dominant species, with *Avicennia marina, Rhizophora apiculata*, and *Rhizophora stylosa* reported consistently across two companion studies conducted at Mundupesisir; one study additionally recorded *Rhizophora mucronata* as a fourth dominant taxon [12], while the other recorded *Acanthus ilicifolius*, a mangrove associate species, in peripheral zones [13]. The mapped extent is bordered by settlement area outside the survey boundary (Fig. 1). Both survey campaigns targeted the same spatial extent.

### 4.2 Equipment and mapping parameters

Both campaigns used the DJI Mavic 3 Enterprise Multispectral (M3E MS) [9], which captures four spectral bands (Green, Red, Red Edge, Near-Infrared) via an integrated sunlight sensor for in-flight irradiance correction. The same mapping parameters were used on both survey dates, so spatial and radiometric conditions stayed consistent between campaigns (Table 3).

**Table 3:** UAV platform, sensor, and mapping specifications, applied consistently for both survey campaigns.

Parameter	Value
UAV platform	DJI Mavic 3 Enterprise Multispectral (M3E MS)
Spectral bands	Green (560 nm), Red (650 nm), Red Edge (730 nm), NIR (860 nm)
Sunlight sensor	Integrated, used for irradiance correction
Flight altitude (AGL)	60 m
Frontal overlap and side overlap	80% and 70%
Flight speed	2.9 m/s
Ground sampling distance (GSD)	2.77 cm/pixel
Global Navigation Satellite System (GNSS) instrument (ground control)	Leica GS18T RTK GNSS (RTK Rover)
Control point count	9 primary points, expanded with additional points
Processing software	Pix4D v4.7.5

### 4.3 Data collection

Flight missions for both campaigns were planned and flown in DJI GS Pro, following the mapping parameters in Table 3. Ground control followed the protocol described in Section 3.3, applied identically on both dates.

### 4.4 Data processing

Multispectral imagery from both campaigns was processed in Pix4D v4.7.5 [10] using the Ag Multispectral template, with identical settings applied to the 5 May 2025 and 12 August 2026 campaigns individually (Table 4). Ground control points were manually marked in at least five images per point during Initial Processing, with the remaining images assisted by Pix4D's automatic GCP detection, to constrain georeferencing. Reflectance calibration relied on in-flight sunlight sensor irradiance measurements rather than a reflectance calibration panel. Initial image alignment used a Keypoints Image Scale of 1 (original image size) with the Aerial Grid image pairs matching strategy. Point cloud densification was performed at half image size with low point density and a minimum of three matches per point, and the 3D textured mesh was generated at medium resolution. Index and reflectance map outputs were generated at 1 × GSD resolution (approximately 2.77 cm/pixel) using Gaussian average downsampling. Single-band reflectance rasters were exported in GeoTIFF format georeferenced in WGS84 / UTM Zone 49S, and RGB orthomosaics were exported and compressed to ECW format.

**Table 4:** Pix4D processing settings applied consistently to both survey campaigns.

Processing stage	Setting	Value	Rationale
Initial Processing	Keypoints Image Scale	1 (original image size)	Full-resolution keypoint matching preserves tie-point accuracy for GCP-based georeferencing.
Point Cloud and Mesh	Point Cloud Densification	1/2 (half image size)	Reduces processing time; the point cloud and mesh are intermediate products used only to support index generation and are not distributed in the repository.
Point Cloud and Mesh	Point Density	Low	Matched to the reduced densification scale; sufficient for the internal surface model used to generate reflectance and index rasters.
Point Cloud and Mesh	Minimum Number of Matches	3	Pix4D's recommended minimum for stable tie-point triangulation without excessive rejection of valid matches over homogeneous canopy areas.
Point Cloud and Mesh	3D Textured Mesh	Medium resolution	Sufficient for visual quality control; not a distributed dataset output.
Index Resolution and Digital Surface Model (DSM)	Resolution	1 × GSD (∼2.77 cm/pixel)	Preserves native sensor resolution in the exported reflectance and index rasters.
Index Resolution and DSM	Downsampling method	Gaussian average	Reduces high-frequency noise in the reflectance rasters while limiting edge artefacts, standard for radiometric index products.

## Limitations

This dataset contains multispectral reflectance and RGB orthomosaic products only; DSM, Digital Terrain Model (DTM), and Canopy Height Model layers are not part of the outputs. The Pix4D template does build an internal surface model while generating the indices, but that raster stays out of the repository. Users needing three-dimensional structural information will need elevation data from another source.

The dataset covers two discrete campaigns approximately 15 months apart (5 May 2025 and 12 August 2026) and does not capture continuous or intra-annual phenological variability. Environmental conditions at the time of acquisition, including tide level and atmospheric haze, may influence reflectance values and should be considered in analyses sensitive to these factors.

Positional accuracy is derived from a network of RTK Rover control points rather than continuous per-image RTK logging; the reported 1D/2D/3D RMS values (Table 2) represent the achieved accuracy of the image-by-image position correction process rather than a direct in-flight RTK measurement for every photograph.

## Ethics Statement

Not applicable.

## CRediT Author Statement

**UA**: Conceptualization, Data Curation and Visualization, Writing; **MLS**: Conceptualization, Resources, Writing, Supervision; **S**: Conceptualization, Resources, Writing, Supervision; **IM**: Data Curation, Writing - Review & Editing; **MRI**: Data Curation, Writing - Review & Editing; **MFH**: Conceptualization, Data Curation and Validation; **IFP**: Conceptualization, Data Curation and Validation; **DFO**: Conceptualization, Data Curation and Validation; **IMR**: Resources, Supervision.

## Declaration of generative AI and AI-assisted technologies in the manuscript preparation process

During the preparation of this manuscript, the author(s) used Claude (Anthropic) to improve the language, grammar, and clarity of the manuscript. The AI tool was not used to generate or interpret scientific data, develop the methodology, or draw scientific conclusions. The authors reviewed and edited all AI-assisted content and take full responsibility for the content of the manuscript.
